# Exercise and Intestinal Barrier Integrity: Molecular Mechanisms and Therapeutic Potential in Gut Health and Disease

**DOI:** 10.3390/biology15181672

**Published:** 2026-09-21

**Authors:** Wenqian Yang, Yuqian Liu, Haitao Wang, Guang Yang

**Affiliations:** 1 Institute of Exercise and Health, Lingnan Normal University, Zhanjiang 524048, China; 2 School of Sports Science, Lingnan Normal University, Zhanjiang 524048, China

**Keywords:** exercise, intestinal barrier, gut microbiota, short-chain fatty acids, intestinal hyperpermeability, inflammation

## Abstract

The intestinal barrier (IB) is a critical interface that maintains internal homeostasis. Its functional compromise—termed intestinal hyperpermeability—permits luminal antigens such as lipopolysaccharide to enter the circulation, triggering metabolic endotoxemia and low-grade systemic inflammation that underpin both intestinal and extra-intestinal diseases. This narrative review was based on a comprehensive literature search of PubMed, Web of Science, and Scopus databases up to July 2026, using keywords related to exercise, intestinal barrier, tight junction proteins, inflammation, oxidative stress, gut microbiota, and short-chain fatty acids. The synthesis indicates that regular moderate-intensity continuous training (30–60 min/session, 3–5 sessions/week) enhances barrier integrity through upregulation of tight junction proteins, suppression of TLR4/NF-κB-driven inflammation, activation of Nrf2-mediated antioxidant defenses, and beneficial remodeling of gut microbiota composition and short-chain fatty acid production. Exercise also induces peripheral–gut crosstalk mediated by myokines and vagal cholinergic signaling. The relationship between exercise volume and barrier function follows a J-shaped dose–response curve, with moderate exercise conferring protection while excessive high-intensity or prolonged exercise transiently increases permeability via splanchnic hypoperfusion and hypoxia-induced epithelial stress. Exercise shows preliminary therapeutic potential as an adjunctive intervention in inflammatory bowel disease, irritable bowel syndrome, and metabolic-associated fatty liver disease, with systemic benefits extending through the gut–liver and gut–brain axes. However, clinical translation remains limited by small sample sizes, short follow-up periods, and heterogeneous protocols. Future research should prioritize large-scale randomized controlled trials, precision exercise prescriptions, and synergistic exercise–probiotic co-interventions to establish definitive clinical guidelines.

## Simple Summary

The gut has a protective lining that keeps harmful substances—such as bacteria and toxins—out of the bloodstream. When this lining weakens, a condition often called “leaky gut,” it can trigger inflammation and contribute to diseases like inflammatory bowel disease, diabetes, obesity, fatty liver disease, and even depression. Exercise appears to be a simple, low-cost way to strengthen this gut barrier. This review explains how exercise works at the molecular level: it tightens the connections between gut cells, reduces inflammation, fights oxidative damage, and promotes the growth of beneficial gut bacteria that produce health-promoting substances. We also describe how muscles and nerves communicate with the gut during physical activity. However, not all exercise is equally beneficial—regular, moderate activity protects the barrier, while very intense or prolonged workouts may temporarily damage it. We discuss how exercise may help manage conditions like irritable bowel syndrome and fatty liver disease, and we point out what we still do not know, such as the best exercise prescription for each person. We suggest that future research should focus on personalised exercise plans and combining exercise with probiotics. In short, exercise is a powerful and accessible tool for gut health, but getting the right dose is key.

## 1. Introduction

The intestinal barrier (IB) is a multilayered defense system orchestrated by the coordinated actions of physical, immunological, microbial, and chemical components. It constitutes the first line of defense between the internal milieu and the external luminal environment, serving the dual role of facilitating efficient nutrient absorption while rigorously restricting the translocation of luminal antigens, including pathogens, toxins, and food-derived macromolecules [1,2]. Among these components, the physical barrier relies primarily on a monolayer of intestinal epithelial cells, which are interconnected by tight junction proteins (TJPs)—including claudins, occludin, and zonula occludens-1 (ZO-1)—that precisely regulate paracellular permeability [3,4]. Beneath the epithelial layer, the lamina propria harbors a diverse array of immune cells responsible for coordinating both innate and adaptive immune responses, collectively forming the immunological barrier [5]. In parallel, the gut microbiota—comprising trillions of microorganisms—further reinforces epithelial integrity through competitive exclusion, production of antimicrobial peptides, and fermentation of dietary components into short-chain fatty acids (SCFAs) [6,7].

Loss of IB integrity—referred to as intestinal hyperpermeability (IH) or “leaky gut”—has been established as a core pathophysiological mechanism underlying a wide spectrum of intestinal and systemic diseases [8,9]. When TJP expression is downregulated or mislocalized, paracellular permeability becomes abnormally elevated, allowing luminal lipopolysaccharide (LPS) and other bacterial products to translocate into the bloodstream, thereby inducing metabolic endotoxemia and low-grade systemic inflammation. This cascade is considered a major driver of inflammatory bowel disease (IBD), irritable bowel syndrome (IBS), celiac disease, and colorectal cancer [1,10]. Of note, barrier dysfunction also contributes to the pathogenesis of non-alcoholic fatty liver disease (NAFLD), obesity, type 2 diabetes mellitus (T2DM), cardiovascular disease, and even neuropsychiatric conditions such as depression and anxiety, via the gut–liver axis and the gut–brain axis [11,12].

The etiology of IB dysfunction is highly complex and multifactorial, encompassing genetic susceptibility, dietary factors such as high-fat and low-fiber intake, chronic psychological stress, gut dysbiosis, and the use of medications including non-steroidal anti-inflammatory drugs [13,14]. In recent years, lifestyle factors—particularly physical activity and exercise—have attracted considerable attention due to their prominent modulatory effects on gut health [15,16]. Exercise induces profound physiological adaptations across multiple systems, including the cardiovascular, metabolic, immune, and neuroendocrine systems [17]. At the intestinal level, exercise can influence splanchnic blood flow, gastrointestinal motility, mucosal immune function, as well as the composition and metabolic activity of the gut microbiota [18]. However, the relationship between exercise and IB integrity is not simply linear: while regular moderate exercise reinforces barrier function, prolonged high-intensity exercise—especially endurance exercise—can transiently increase intestinal permeability, a phenomenon termed exercise-induced intestinal hyperpermeability (EIIH) [18,19].

A deep understanding of the molecular mechanisms by which exercise modulates IB integrity is essential for the development of exercise prescriptions targeting gut health. Recent studies have progressively elucidated the complex signaling pathways involved—exercise has been shown to: (i) activate the AMP-activated protein kinase (AMPK) pathway, thereby promoting TJP assembly and stability [20]; (ii) suppress the Toll-like receptor 4/nuclear factor-κB (TLR4/NF-κB) inflammatory cascade, reducing the production of pro-inflammatory cytokines such as tumor necrosis factor-α (TNF-α), interleukin-6 (IL-6), and interleukin-1β (IL-1β) [21]; (iii) upregulate the nuclear factor erythroid 2-related factor 2 (Nrf2)-mediated antioxidant response, protecting intestinal epithelial cells from oxidative stress-induced damage [22]; and (iv) induce beneficial shifts in gut microbiota composition, increasing the abundance of SCFA-producing bacteria, including Faecalibacterium prausnitzii and Roseburia hominis. Notably, butyrate—a key metabolite produced by these bacteria—enhances tight junction integrity through inhibition of histone deacetylases (HDACs) and activation of the AMPK pathway [23,24].

Beyond these direct effects on the intestine, exercise also facilitates bidirectional communication between peripheral organs and the intestinal epithelium. Contracting skeletal muscle releases myokines—including irisin, interleukin-6 (IL-6), and brain-derived neurotrophic factor (BDNF)—which signal to the gut via the circulation to modulate immune and epithelial functions [25,26]. Notably, irisin has been shown to attenuate intestinal inflammation and improve IB integrity in experimental colitis models [27]. In addition, the autonomic nervous system (ANS), particularly the vagus nerve, mediates exercise-induced anti-inflammatory effects on the gut through the cholinergic anti-inflammatory pathway [28].

Despite considerable progress made in recent years, several key scientific questions in this field remain to be fully elucidated. Most studies have been based on animal models or small-scale human cohorts, and large-scale randomized controlled trials (RCTs) evaluating the dose–response relationship between exercise and IB integrity remain scarce [29]. At present, no consensus has been reached regarding which exercise modality—moderate-intensity continuous training (MICT), resistance training (RT), or high-intensity interval training (HIIT)—or what intensity, frequency, and duration are most effective for maintaining or restoring IB function [30]. Furthermore, individual factors such as baseline gut microbiota composition, genetic background, age, sex, and disease status may influence the responsiveness to exercise, highlighting the need for approaches grounded in precision exercise medicine [15,31].

Despite the accumulating evidence reviewed above, several critical knowledge gaps remain: the comparative efficacy of different exercise modalities on barrier outcomes, the causal hierarchy among the proposed molecular pathways, and the translational potential of exercise as a stand-alone or adjunctive therapy in patients with established intestinal diseases. The present review aims to address these gaps by comprehensively synthesizing mechanistic, physiological, and clinical evidence on exercise–barrier interactions, and by deriving evidence-based recommendations for future research and clinical practice.

This review provides a comprehensive narrative synthesis of the molecular, neural, and physiological mechanisms by which exercise regulates intestinal barrier integrity. Specifically, we examine five major interconnected pathways: (i) the regulation of tight junction proteins via AMPK/MLCK and HSP70 signaling; (ii) the suppression of TLR4/NF-κB-driven intestinal inflammation; (iii) the activation of Nrf2-mediated antioxidant defenses; (iv) the remodeling of gut microbiota composition and short-chain fatty acid production; and (v) the peripheral–gut crosstalk mediated by myokines and autonomic nervous system signals. We then evaluate the differential effects of various exercise modalities, intensities, and durations on barrier outcomes, and articulate the J-shaped dose–response relationship between exercise volume and intestinal permeability. The therapeutic potential of exercise as an adjunctive intervention in inflammatory bowel disease, irritable bowel syndrome, and metabolic-associated fatty liver disease is critically assessed. We further explore the downstream systemic benefits conferred by improved barrier function via the gut–liver axis and the gut–brain axis, linking intestinal barrier integrity to metabolic and neuropsychiatric health. Finally, we identify key limitations in the current evidence base and outline future directions, including precision exercise medicine, multi-omics integration, and combined exercise–probiotic co-interventions—emerging strategies that require further empirical validation before clinical translation can be considered—with the goal of providing a theoretical foundation for the development of IB-targeted exercise prescriptions.

This narrative review was conducted following the scale for the assessment of narrative review articles (SANRA) guidelines. A comprehensive literature search was performed in PubMed, Web of Science, and Scopus databases for studies published from database inception to July 2026. The search strategy combined keywords related to exercise (e.g., “exercise,” “physical activity,” “training,” “MICT,” “HIIT,” “resistance training”), intestinal barrier function (e.g., “intestinal barrier,” “gut barrier,” “intestinal permeability,” “tight junction,” “leaky gut”), and molecular mechanisms (e.g., “AMPK,” “TLR4/NF-κB,” “Nrf2,” “gut microbiota,” “short-chain fatty acids,” “myokines”). Reference lists of relevant reviews and included articles were manually screened to identify additional eligible studies. Only peer-reviewed English-language articles were considered. Given the narrative nature of this review, no formal quality assessment or quantitative meta-analysis was performed. Instead, we critically appraised and synthesized the available evidence to provide an integrated overview of the field.

## 2. Exercise-Induced Regulation of Intestinal Barrier Integrity: Molecular Mechanisms

The intestinal barrier (IB) is a dynamic structure capable of responding to a variety of physiological and pathological stimuli, including exercise. Accumulating evidence indicates that exercise modulates IB integrity through multiple interconnected molecular pathways [20,32]. These pathways encompass the regulation of tight junction proteins (TJPs), the modulation of inflammatory signaling cascades, the activation of antioxidant defense systems, the remodeling of gut microbiota composition and its metabolic products, and the actions of myokines and neuroendocrine factors that mediate bidirectional communication between peripheral organs and the gut [25,26]. This section provides a comprehensive narrative review of the current understanding of these molecular mechanisms, with a particular focus on signaling pathways that have been well validated in both animal models and human subjects [20,32].

### 2.1. Exercise Regulation of Tight Junction Proteins

Tight junctions (TJs) are multiprotein complexes localized to the apical junction complex of intestinal epithelial cells. They form a continuous intercellular seal that governs paracellular permeability [4,33]. The core TJPs include transmembrane proteins such as claudins, occludin, and tricellulin, as well as cytoplasmic scaffolding proteins including ZO-1, ZO-2, and ZO-3, with the latter connecting the transmembrane proteins to the actin cytoskeleton [3,4]. The expression levels, phosphorylation status, and subcellular localization of these proteins collectively determine the degree of intestinal permeability. Under various pathological conditions, downregulation or redistribution of ZO-1, occludin, and claudin-1 are considered hallmarks of IH [1,9,34].

A wealth of evidence indicates that regular moderate exercise upregulates TJP expression, thereby reinforcing IB integrity [20,32,35]. In high-fat-diet-induced obese mouse models, 12 weeks of voluntary wheel running successfully restored ZO-1 and occludin expression levels in the small intestine and colon, accompanied by reductions in serum LPS levels and improvements in metabolic parameters [36]. In human studies, a randomized controlled trial (RCT) in sedentary overweight adults demonstrated that six weeks of MICT increased colonic ZO-1 mRNA expression and reduced the lactulose/mannitol ratio—a gold-standard measure of intestinal permeability [37].

The molecular pathways through which exercise upregulates TJPs have been partially elucidated. AMPK plays a pivotal role in this process—it is activated by exercise-induced increases in the AMP/ATP ratio and by calcium/calmodulin-dependent protein kinase kinase (CaMKK) signaling [20]. Upon activation, AMPK directly phosphorylates and stabilizes TJPs on one hand, while on the other, it suppresses the activity of the myosin light chain kinase (MLCK) pathway—a key disruptor of tight junction integrity that promotes paracellular channel opening through phosphorylation of myosin light chain (MLC) and subsequent cytoskeletal contraction [32]. Previous studies have demonstrated that exercise-induced AMPK activation inhibits MLCK activity and MLC phosphorylation in intestinal epithelial cells, thereby preserving TJ integrity [38]. This mechanism has been validated at the cellular level: in human colon adenocarcinoma (Caco-2) cell monolayers, pharmacological activation of AMPK with AICAR increases transepithelial electrical resistance (TEER) and decreases permeability, recapitulating the effects observed with exercise [20].

In addition to AMPK, heat shock protein 70 (HSP70) also participates in this regulatory process. HSP70 is induced by exercise-associated heat stress and oxidative stress, and serves as a molecular chaperone that facilitates the proper folding and assembly of TJPs while protecting them from degradation [32,39]. In rat models of heat stress, moderate exercise preconditioning upregulates HSP70 expression in the intestinal epithelium, thereby preventing the loss of ZO-1 and occludin induced by subsequent severe heat stress [32]. In cultured Caco-2 intestinal epithelial cells, HSP70 overexpression increases TEER and reduces permeability, whereas HSP70 knockdown produces the opposite effect [32], suggesting that exercise-induced HSP70 expression contributes to the stabilization of TJ complexes.

Of note, excessive or prolonged strenuous exercise produces the opposite effect—downregulating TJPs and exacerbating IH. In a study of marathon runners, plasma levels of intestinal fatty acid-binding protein (I-FABP), a marker of intestinal epithelial cell injury, were significantly elevated immediately post-race, and this elevation correlated with decreased serum occludin levels [19]. In animal experiments, exhaustive treadmill exercise similarly reduced ZO-1 and claudin-1 expression in the small intestine, accompanied by increased LPS translocation [40]. This deleterious effect is likely mediated by splanchnic ischemia–reperfusion injury, which in turn triggers the generation of reactive oxygen species (ROS) and activation of matrix metalloproteinases (MMPs), ultimately leading to TJP degradation [40].

Collectively, moderate exercise enhances IB integrity by upregulating the expression of tight junction proteins and stabilizing their assembly via the AMPK/MLCK and HSP70 pathways, whereas exhaustive exercise produces the opposite effect. These divergent outcomes likely depend on exercise intensity, duration, and the individual’s training status [38].

Of note, the evidence supporting the AMPK/MLCK and HSP70 pathways is predominantly derived from rodent models and cultured epithelial cell lines [20,32]. Human data remain sparse, with most supportive evidence coming from indirect measurements such as circulating biomarkers or permeability ratios rather than direct intestinal tissue analyses [37]. The extrapolation of these mechanistic findings to humans, therefore, warrants caution, and future studies incorporating endoscopic biopsy sampling in exercise intervention contexts are needed to confirm the translational relevance of these pathways (Table 1).

### 2.2. Exercise and Intestinal Inflammation: The TLR4/NF-κB Pathway

Chronic low-grade inflammation is both a cause and a consequence of IB injury. Studies have shown that pro-inflammatory cytokines such as TNF-α, IL-1β, and IL-6 can increase intestinal permeability by disrupting TJPs and inducing epithelial cell apoptosis [1,9,34]. In this process, the TLR4/NF-κB signaling pathway serves as a central mediator of intestinal inflammation. Activation of TLR4 by LPS triggers a cascade of events that promotes NF-κB nuclear translocation and initiates the transcription of pro-inflammatory genes [11]. TLR4 signaling activates downstream inflammatory responses via two distinct pathways—the myeloid differentiation factor 88 (MyD88)-dependent pathway and the TRAM/TRIF-dependent pathway [41].

Exercise exerts potent anti-inflammatory effects, a substantial portion of which is mediated through suppression of the TLR4/NF-κB pathway [21]. Regular moderate exercise reduces circulating levels of inflammatory markers, including C-reactive protein (CRP), TNF-α, and IL-6, in both healthy individuals and patients with chronic inflammatory diseases [21]. At the intestinal level, multiple studies have demonstrated that exercise training suppresses TLR4/NF-κB signaling in intestinal epithelial cells and macrophages [42].

In high-fat-diet-induced obese mouse models, 12 weeks of voluntary wheel running significantly reduced colonic expression of TLR4, MyD88, and phosphorylated NF-κB p65, accompanied by decreased levels of TNF-α and IL-1β in the intestinal mucosa [36]. These changes were closely associated with improved barrier function, as evidenced by reduced LPS translocation and increased expression of ZO-1 and occludin [36]. Similar observations were made in the chronic unpredictable mild stress (CUMS) rat model—which induces intestinal inflammation and IB injury—in which 8 weeks of treadmill exercise significantly reduced colonic TLR4 expression and NF-κB activation, and this effect correlated with decreased pro-inflammatory cytokine levels and restoration of TJP expression [43].

In human studies, a randomized controlled trial (RCT) in sedentary overweight adults demonstrated that six weeks of MICT reduced TLR4 expression in peripheral blood mononuclear cells, along with decreased plasma levels of TNF-α and IL-6 [44]. Although direct evidence that exercise downregulates intestinal TLR4/NF-κB signaling in humans remains insufficient, the systemic anti-inflammatory effects of exercise are well-established and are highly likely to extend to the intestinal mucosal level [21].

The mechanisms by which exercise suppresses TLR4/NF-κB signaling are multifaceted. First, exercise reduces the availability of TLR4 ligands by improving IB integrity and lowering circulating LPS levels [11]. Second, exercise induces the release of anti-inflammatory cytokines, including interleukin-10 (IL-10) and interleukin-1 receptor antagonist (IL-1RA), which in turn inhibit NF-κB activation [21]. Third, exercise activates the vagus nerve-mediated cholinergic anti-inflammatory pathway—efferent vagal fibers release acetylcholine, which binds to α7 nicotinic acetylcholine receptors (α7nAChRs) on macrophages and intestinal epithelial cells, thereby suppressing NF-κB nuclear translocation and reducing pro-inflammatory cytokine production [28]. In animal models, vagotomy abolishes the protective effects of exercise against intestinal inflammation, further confirming the importance of this neural pathway [28].

Of note, acute high-intensity or prolonged exhaustive exercise may produce a diametrically opposite effect—transiently activating the TLR4/NF-κB pathway and exacerbating intestinal inflammation. This is of particular concern in endurance athletes, in whom strenuous exercise is often accompanied by elevated plasma levels of IL-6, TNF-α, and lipopolysaccharide binding protein (LBP), suggesting activation of gut-derived inflammatory responses [45]. Nevertheless, these effects are typically transient and are followed by an anti-inflammatory recovery phase. Following regular training, the pro-inflammatory response to a given exercise workload is attenuated, reflecting the establishment of adaptive immune tolerance [21].

In summary, regular moderate exercise alleviates intestinal inflammation by suppressing the TLR4/NF-κB pathway, thereby contributing to the maintenance of IB integrity, whereas acute exhaustive exercise transiently activates this pathway, underscoring the importance of appropriately prescribed exercise regimens [38,45].

It is important to recognize that while the anti-inflammatory effects of exercise on the TLR4/NF-κB pathway are well established in animal models [36,42], the direct evidence in humans is largely circumstantial, relying primarily on reductions in circulating inflammatory markers rather than direct assessment of intestinal mucosal NF-κB activity [21,44]. The strength of this evidence is therefore moderate at best, and causal inferences regarding the pathway-specific mechanisms operative in the human intestine remain provisional.

Chronic exhaustive exercise has also been associated with alterations in the mucosal immune system, including reduced secretory IgA levels and altered lymphocyte composition in mesenteric lymph nodes, which may contribute to impaired epithelial barrier integrity over time [46].

### 2.3. Exercise and Intestinal Oxidative Stress: The Nrf2 Pathway

Reactive oxygen species (ROS) play a double-edged sword role in intestinal barrier function. At low-to-moderate levels, ROS act as signaling molecules that activate adaptive stress responses; however, when ROS production becomes excessive, the endogenous antioxidant defense system is rapidly overwhelmed, leading to lipid peroxidation, protein oxidation, and DNA damage, ultimately culminating in epithelial cell death and disruption of IB integrity [9,22]. Intestinal epithelial cells are particularly susceptible to oxidative stress due to their high metabolic rate and continuous exposure to luminal oxidants [9].

Nrf2 is a core transcription factor that regulates the expression of multiple antioxidant and cytoprotective genes [47]. Under basal conditions, Nrf2 is sequestered in the cytoplasm and targeted for ubiquitin-dependent degradation through its binding to its inhibitor, Kelch-like ECH-associated protein 1 (Keap1) [22]. When cells are exposed to oxidative stress or electrophilic agents, key cysteine residues in Keap1 undergo chemical modification, triggering the release and nuclear translocation of Nrf2 [48]. Upon entering the nucleus, Nrf2 heterodimerizes with small Maf proteins and binds to the antioxidant response element (ARE) in the promoter regions of target genes, initiating the transcription of phase II detoxifying enzymes and antioxidant proteins, including heme oxygenase-1 (HO-1), NAD(P)H quinone oxidoreductase 1 (NQO1), superoxide dismutase (SOD), catalase (CAT), and glutathione peroxidase (GPx) [22].

Studies have confirmed that exercise activates the Nrf2 pathway in multiple tissues, including skeletal muscle, heart, liver, and brain, and this activation is recognized as one of the key mechanisms underlying the beneficial effects of exercise against oxidative stress-related diseases [22]. In the intestinal context, accumulating evidence indicates that regular moderate exercise upregulates Nrf2 signaling in intestinal epithelial cells, thereby enhancing antioxidant capacity and protecting the IB against oxidative damage [22].

In a rat model of intestinal ischemia–reperfusion injury—which is characterized by extensive ROS production and severe IB disruption—four weeks of treadmill exercise preconditioning significantly attenuated mucosal damage, preserved TJP expression, and reduced intestinal permeability [40]. These protective effects were closely correlated with increased Nrf2 nuclear translocation, upregulation of HO-1 and NQO1, and decreased levels of malondialdehyde (MDA) [22]. In Nrf2 knockout mice, the protective effects of exercise against ischemia–reperfusion-induced intestinal injury were completely abolished, further confirming the critical role of Nrf2 in this process [22].

Similar results have been observed in models of chronic inflammatory stress. In high-fat diet-fed mice, 12 weeks of voluntary wheel running increased colonic Nrf2 activity, elevated the expression levels of SOD, CAT, and GPx, and reduced oxidative stress markers, including 8-hydroxy-2′-deoxyguanosine (8-OHdG) and 4-hydroxynonenal (4-HNE) [49]. These changes were accompanied by improved IB integrity and reduced LPS translocation [49]. In the CUMS rat model, 8 weeks of treadmill exercise similarly upregulated colonic expression of Nrf2 and its downstream target HO-1, reduced ROS levels, and restored ZO-1 and occludin expression [43].

The mechanisms by which exercise activates Nrf2 in intestinal epithelial cells involve multiple upstream signaling pathways. First, exercise induces a moderate and transient increase in ROS production in intestinal epithelial cells—as well as in skeletal muscle and other tissues—which serves precisely as a signal for Nrf2 activation, a concept termed “oxidative preconditioning” or “hormesis” [22]. This transient ROS burst modifies sulfhydryl groups on Keap1, thereby promoting the release of Nrf2. Second, exercise activates AMPK, which can directly phosphorylate Nrf2 at serine 550, enhancing its stability and transcriptional activity [20]. Third, exercise-induced activation of the phosphoinositide 3-kinase (PI3K)/protein kinase B (Akt) pathway also contributes to the nuclear accumulation of Nrf2 [22].

Of note, acute exhaustive exercise or prolonged strenuous exercise may overwhelm the Nrf2-mediated antioxidant defense system, resulting in net oxidative damage. In a study of marathon runners, plasma levels of oxidative stress markers, including MDA and protein carbonyls, were significantly elevated immediately post-race and correlated with increased intestinal permeability [19]. In animal experiments, a single bout of exhaustive treadmill exercise reduced nuclear Nrf2 levels in the small intestine, decreased HO-1 and NQO1 expression, and rendered the animals more susceptible to subsequent oxidative challenges [32]. These findings suggest that although regular moderate exercise strengthens the Nrf2 antioxidant system, excessive exercise depletes this defensive capacity, tilting the balance toward oxidative injury [38].

In humans, direct evidence for exercise-induced Nrf2 activation in the intestine remains limited; however, indirect evidence provides some support. In a randomized controlled trial (RCT) involving sedentary overweight adults, six weeks of MICT increased plasma total antioxidant capacity and reduced urinary F2-isoprostane levels, a marker of systemic oxidative stress [37]. Although intestinal tissue was not directly sampled, the improvement in intestinal permeability (as measured by the lactulose/mannitol ratio) was positively correlated with total antioxidant capacity, suggesting that a link between systemic antioxidant status and IB integrity may exist [37].

In summary, regular moderate exercise activates the Nrf2/ARE pathway, upregulates antioxidant enzymes, and alleviates oxidation-mediated IB injury—a key mechanism by which exercise protects the IB [22]. Of note, there is crosstalk between the Nrf2 pathway and the TLR4/NF-κB pathway, and the anti-inflammatory and antioxidant effects of exercise are achieved synergistically through these two pathways [47].

It should be noted that the Nrf2-mediated antioxidant pathway is among the best-characterized exercise-responsive mechanisms at the molecular level, with robust evidence from both in vitro and in vivo animal studies [22,40]. However, direct evidence for Nrf2 activation in the human intestine remains limited to indirect associations between systemic antioxidant capacity and permeability indices [37]. Whether the same regulatory dynamics observed in rodent models operate similarly in the human gut mucosa, particularly under pathological conditions, remains to be definitively established.

### 2.4. Exercise, Gut Microbiota, and Short-Chain Fatty Acids

The gut microbiota (GM) is a complex ecosystem composed of trillions of microorganisms colonizing the gastrointestinal tract and serves as a key mediator linking exercise and the IB [15,16]. The composition and function of the GM are highly responsive to lifestyle factors, and numerous studies have confirmed that exercise can modulate microbial diversity, species composition, and metabolic function [15,16]. Short-chain fatty acids (SCFAs) are core metabolic products generated by microbial fermentation of dietary fiber and play a critical role in maintaining epithelial homeostasis and regulating immune responses [5,7].

#### 2.4.1. Exercise-Induced Remodeling of Gut Microbiota Composition

Numerous studies have confirmed that regular physical activity reshapes gut microbiota composition across diverse populations, including healthy individuals, athletes, and patients with metabolic disorders [23,50]. A systematic review and meta-analysis encompassing 19 studies with 1062 participants demonstrated that exercise significantly increased the Shannon index of α-diversity in obese individuals and those with type 2 diabetes, and consistently observed enrichment of butyrate-producing bacteria (notably Roseburia and Faecalibacterium prausnitzii) as well as Akkermansia muciniphila [50]. Similar conclusions were drawn from studies in elite athletes [23]. Interventional studies have further confirmed that structured exercise programs increase the abundance of beneficial genera, including Faecalibacterium, Veillonella, Lachnospira, and Bifidobacterium, and that these changes are closely associated with anti-inflammatory effects and improvements in metabolic profiles [15,23].

In obese populations, exercise also significantly modulates gut microbiota composition [51]. The gut microbiota of athletes exhibits a unique compositional profile that may serve as a biomarker of exercise adaptations [52,53]. Furthermore, the interaction between exercise and the gut microbiota is influenced by exercise intensity, type, and duration [54].

#### 2.4.2. Short-Chain Fatty Acids as Key Mediators of Exercise-Induced Barrier Protection

Short-chain fatty acids exert protective effects on intestinal barrier integrity through multiple mechanisms [7]. Among these, butyrate serves as the primary energy source for colonocytes, promoting their proliferation and differentiation, while simultaneously enhancing tight junction assembly through activation of AMPK and inhibition of histone deacetylases [5,7]. Propionate and acetate, in turn, play a primary role in regulating immune responses and promoting mucus secretion [7].

Of note, the effects of exercise on SCFA production exhibit a clear intensity-dependent pattern [55]. A pooled analysis of three randomized controlled trials (comprising 113 patients with metabolic syndrome) evaluated the effects of 12 weeks of high-intensity interval training versus moderate-intensity interval training combined with a single session of resistance training [55]. The results demonstrated that only the high-intensity interval training group showed a significant increase in total fecal SCFAs (by 30%), with increases of 27% for acetate, 28% for propionate, and 43% for butyrate [55]. Mean blood lactate concentration during training was strongly correlated with the increase in SCFAs (r = 0.68, *p* < 0.001), suggesting that lactate generated during high-intensity exercise may serve as a precursor or signaling molecule for microbial SCFA production [55]. This lactate-mediated microbial adaptation represents a novel mechanism linking exercise intensity to metabolic benefits in the gut [55].

Short-chain fatty acids—particularly butyrate—exert anti-inflammatory effects by activating G protein-coupled receptors and inhibiting histone deacetylases, while also providing energy substrates for colonocytes and for muscles during exercise [5,7]. SCFAs synthesized by the gut microbiota have been shown to confer significant improvements in exercise performance [56]. Collectively, the interplay among exercise, the gut microbiota, and SCFAs constitutes one of the key mechanisms underlying the health-promoting effects of exercise [57].

It is important to reconcile the observation that butyrate increases are observed predominantly with high-intensity exercise with the broader recommendation that moderate-intensity exercise is preferable for overall gut barrier health. These two claims are not contradictory, as they derive from distinct study contexts and address different outcome measures. The finding that butyrate production increases following high-intensity interval training originates from a 12-week RCT in patients with metabolic syndrome, in which total fecal SCFAs and absolute butyrate content were measured, and in which the HIIT protocol was compared against MICT combined with resistance training [55]. The strong correlation between blood lactate concentration and SCFA elevation observed in that study suggests that lactate accumulation during high-intensity exercise may serve as a precursor substrate for microbial butyrate synthesis [55]. By contrast, the recommendation that moderate-intensity exercise is preferable for gut health is drawn from a broader synthesis of the literature evaluating comprehensive barrier outcomes—including tight junction protein expression, intestinal permeability, systemic low-grade inflammation, and the intestinal injury marker I-FABP [20,29,37]. Acute high-intensity exercise sessions are known to induce splanchnic hypoperfusion and transient intestinal hyperpermeability, whereas regular moderate-intensity exercise stably improves all barrier indices without acute injury risk [18,40,58]. Thus, while chronic HIIT adaptation may enhance gut-protective metabolites, acute high-intensity bouts transiently compromise barrier integrity. These two findings are complementary rather than contradictory: moderate-intensity exercise provides robust barrier protection through multiple pathways without acute risk, whereas the SCFA-mediated benefits of high-intensity exercise must be weighed against its potential transient barrier-disrupting effects, with applicability depending on population, tolerance, and clinical context.

The interplay between exercise and the gut microbiota also raises the possibility of synergistic co-interventions combining exercise with probiotics or prebiotics, a topic we address in the context of future research directions (Section 6.3).

#### 2.4.3. Gut–Muscle Axis and Broader Implications

The gut microbiota is not merely a passive responder to exercise; it also actively participates in modulating host exercise capacity and metabolic adaptations [15]. The concept of the “gut–muscle axis” was proposed to describe the bidirectional communication between the gut microbiome and skeletal muscle, with short-chain fatty acids serving as key signaling molecules in this crosstalk [15]. The enteric nervous system integrates mechanical, immune, and microbial signals in real time, modulating gastrointestinal motility, barrier function, and microbial ecology during exercise, while transmitting gut-derived signals to muscles and the brain via neural and humoral routes [16].

The exercise-induced changes in gut microbiota composition and SCFA production also exert systemic effects that extend beyond the gut itself. Through the gut–brain axis, SCFAs can influence neurotransmitter synthesis and the progression of neuroinflammatory processes [12]; through the gut–liver axis, the reduction in LPS translocation resulting from improved barrier integrity can alleviate hepatic inflammation and steatosis [59].

Among the mechanisms discussed, the gut microbiota–SCFA pathway is supported by relatively stronger human evidence, with consistent findings across multiple cohort studies and small-scale intervention trials [23,50,55]. Nevertheless, the majority of these studies are correlative in nature, and the extent to which exercise-induced changes in microbial composition directly mediate improvements in barrier function, as opposed to being merely associated with them, requires further elucidation through mechanistic intervention studies, including fecal microbiota transplantation and metabolomic profiling, (Table 2).

### 2.5. Exercise-Induced “Peripheral–Gut Dialogue”: Myokines and Neural Signals

The beneficial effects of exercise on intestinal barrier integrity are not confined to local adaptations within the gut itself. Rather, exercise triggers a systemic response involving signaling molecules released by contracting skeletal muscle and the activation of autonomic neural pathways, all of which collectively constitute a mechanism of “peripheral–gut dialogue” [25,26].

#### 2.5.1. Myokines as Humoral Mediators

Skeletal muscle functions as an endocrine organ, secreting hundreds of bioactive factors during contraction—collectively termed myokines [25]. The bidirectional relationship between myokines and gut microbiota has recently emerged as a novel component of the endocrine muscle–gut axis, with potential implications for metabolic health [63]. Irisin is a myokine cleaved from FNDC5 and is released into circulation during exercise [26]. Emerging evidence indicates that irisin exerts protective effects on the gut: in dextran sulfate sodium (DSS)-induced colitis mouse models, systemic administration of irisin attenuates colonic inflammation and preserves the expression of tight junction proteins [27]. In Caco-2 cell monolayers, recombinant irisin treatment increases transepithelial electrical resistance and reduces the flux of FITC-dextran [58]. Interleukin-6 is another myokine released in large amounts during exercise—exercise-induced IL-6 exerts its anti-inflammatory effects by stimulating the production of IL-10 and IL-1RA [25]. In a rat model of intestinal ischemia–reperfusion injury, exercise preconditioning increases serum IL-6 levels, whereas neutralization of IL-6 abolishes the protective effect of exercise on barrier function [40]. Brain-derived neurotrophic factor is also produced by skeletal muscle and can act on the gut either via the circulation or through vagal afferent signals, thereby reducing intestinal permeability [58].

#### 2.5.2. Autonomic Nervous System and the Cholinergic Anti-Inflammatory Pathway

The vagus nerve plays a critical role in mediating the anti-inflammatory effects of exercise on the gut [28]. During exercise, vagal tone changes, and acetylcholine released by efferent vagal fibers activates α7 nicotinic acetylcholine receptors (α7nAChRs) on intestinal macrophages and epithelial cells—a mechanism termed the “cholinergic anti-inflammatory pathway” [28]. Activation of α7nAChRs suppresses the TLR4/NF-κB pathway, thereby reducing pro-inflammatory cytokine production and preserving tight junction integrity [28]. In a rat model of exercise-induced heat stress, vagotomy abolishes the protective effects of exercise preconditioning against intestinal barrier disruption [28].

#### 2.5.3. Integration

**Figure 1:**
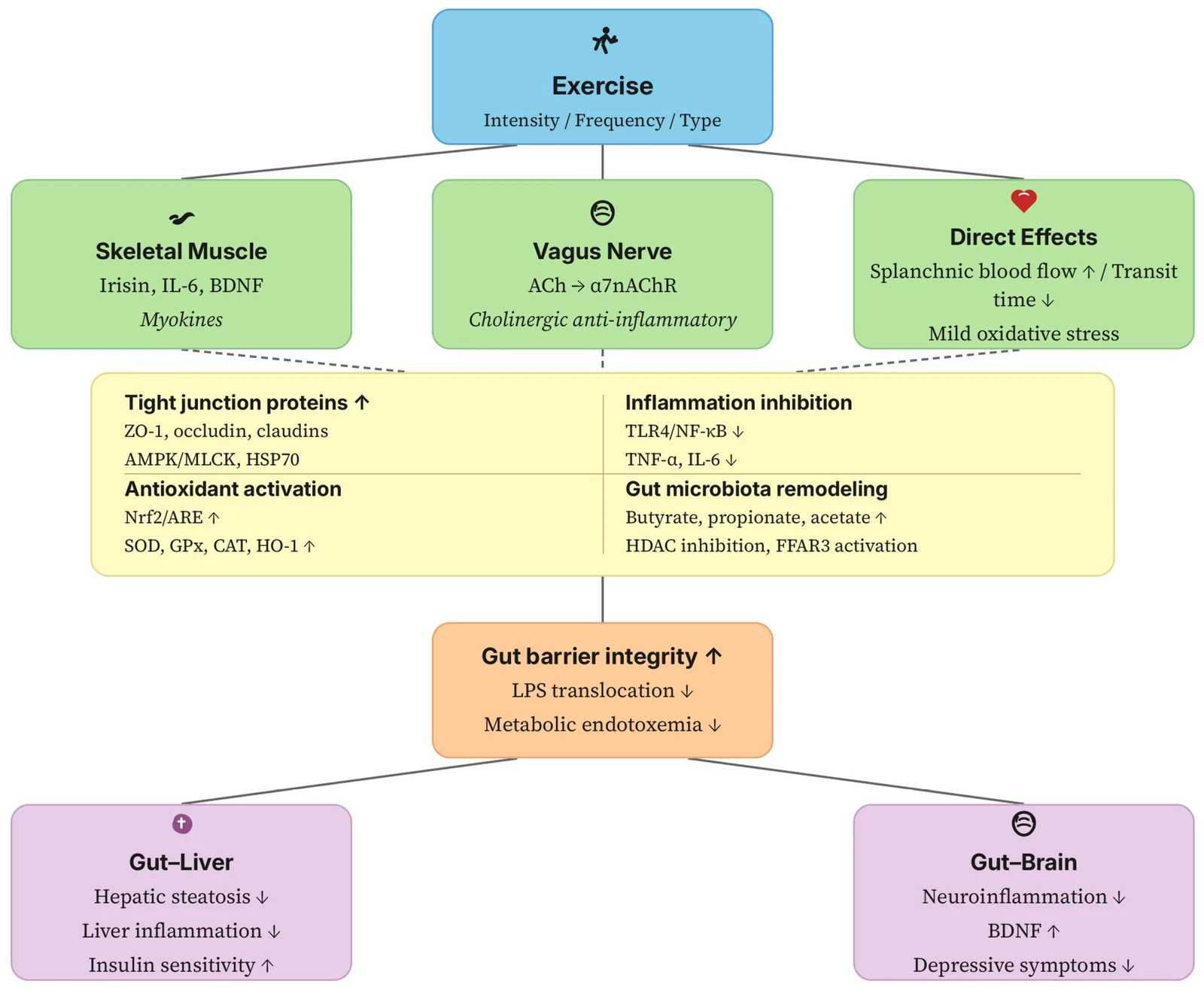
Integrated Schematic of Multipathway Mechanisms by Which Exercise Regulates Intestinal Barrier Integrity. ↑ increase, ↓ decrease; solid lines: direct, dashed lines: indirect. (Schematic diagram illustrating the integrated molecular mechanisms by which exercise enhances intestinal barrier integrity. Exercise triggers peripheral signals from three sources: skeletal muscle (myokines: irisin, IL-6, BDNF), the autonomic nervous system (vagal cholinergic pathway), and direct mechanical/metabolic effects (splanchnic blood flow, transit time, mild oxidative stress). These signals converge on the intestinal epithelium and act through the following pathways: (1) upregulation of tight junction proteins (ZO-1, occludin, claudins) via the AMPK/MLCK and HSP70 pathways; (2) suppression of TLR4/NF-κB-driven inflammatory responses; (3) activation of the Nrf2-mediated antioxidant defense system (SOD, GPx, CAT, HO-1); and (4) remodeling of the gut microbiota to increase short-chain fatty acid (acetate, propionate, butyrate) production. SCFAs, in turn, further reinforce tight junctions, inhibit histone deacetylases, and activate free fatty acid receptor 3 (FFAR3)-dependent secretion of glucagon-like peptide-1 (GLP-1). These mechanisms act synergistically to reduce intestinal permeability, prevent lipopolysaccharide translocation, and avert gut-derived systemic inflammation. Subsequently, the gut–liver axis and gut–brain axis translate these local benefits into alleviation of hepatic steatosis/inflammation and improvement in neurobehavioral outcomes. Source: The authors.)

The peripheral–gut dialogue during exercise involves a complex, multilayered regulatory network [25,26]. Contracting muscles release myokines that act directly on intestinal epithelial cells and immune cells. Concurrently, afferent signals from mechanoreceptors and chemoreceptors in muscles and the gut activate the vagal reflex. Efferent vagal fibers release acetylcholine, which binds to α7nAChRs to suppress inflammation [28]. This integrated system ensures that the gut can maintain its fundamental barrier function while accommodating the systemic demands of exercise [17] (Figure 1).

### 2.6. Dose–Response Relationship Between Exercise and Intestinal Barrier: The J-Shaped Curve

**Figure 2:**
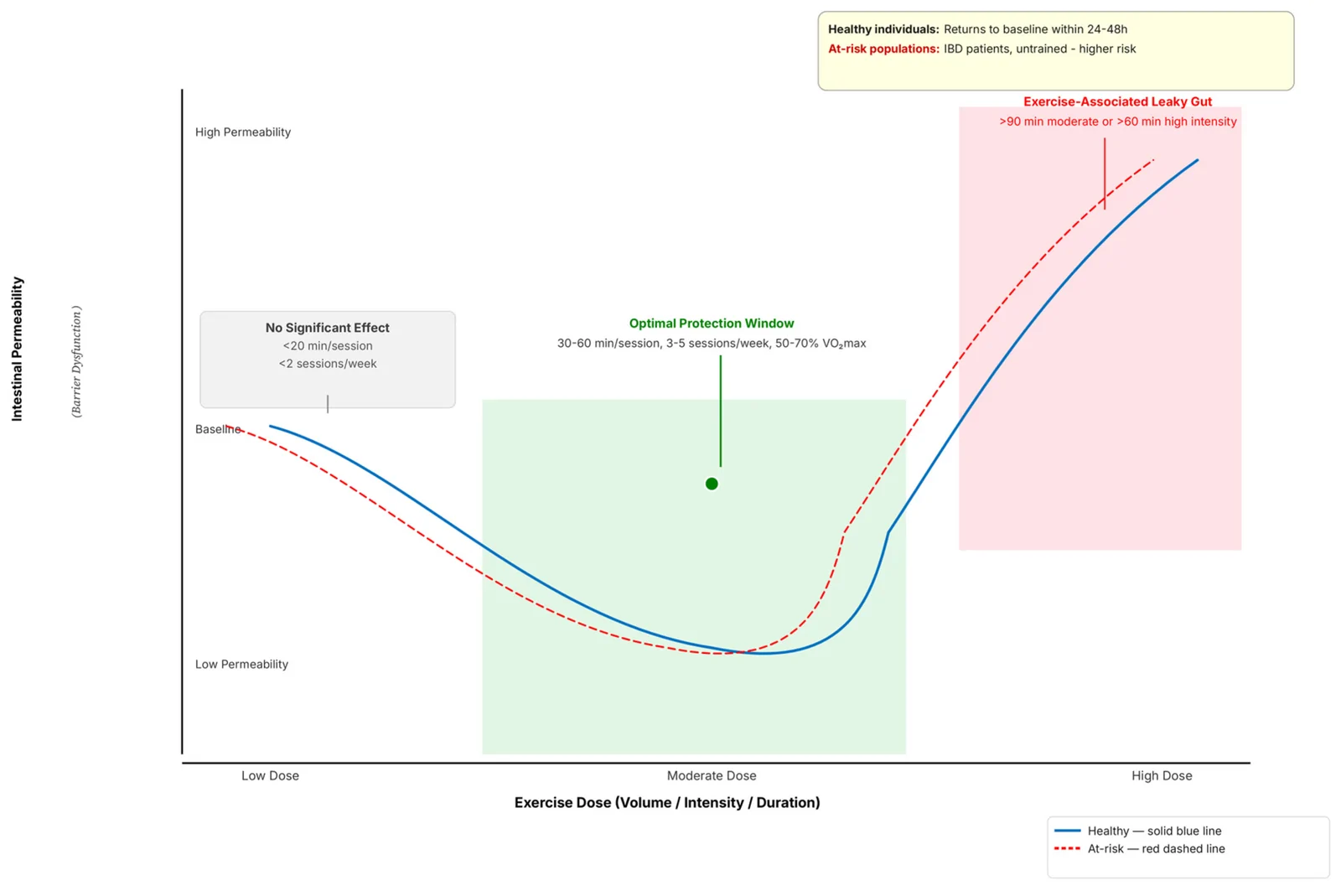
J-Shaped Dose–Response Curve Between Exercise Volume and Intestinal Barrier Integrity.(Illustration of the J-shaped relationship between exercise dose and intestinal barrier function. Low-dose exercise—for example, <20 min per session, <2 sessions per week, low intensity—yields only marginal improvements. Moderate-dose exercise—30–60 min per session, 3–5 sessions per week, 50–70% of maximal oxygen uptake (VO_2_max)—provides optimal protective effects, including reduced intestinal permeability, enhanced tight junction expression, and decreased systemic lipopolysaccharide levels. High-dose exercise—>90 min per session at moderate intensity, or >60 min at high intensity, particularly in hot environments—transiently increases permeability (i.e., “exercise-induced intestinal hyperpermeability”), accompanied by elevations in I-FABP, lipopolysaccharide, and inflammatory markers. In healthy individuals, permeability returns to baseline within 24–48 h; however, repeated excessive exercise without adequate recovery may pose a risk to susceptible populations, such as patients with inflammatory bowel disease and untrained individuals. The “safety window” for gut health is indicated by the green shaded area. It should be noted that the SCFA-mediated benefits of high-intensity exercise, as reported in some studies, reflect chronic adaptive responses in specific populations (e.g., patients with metabolic syndrome) and do not negate the transient barrier-disrupting effects of acute high-intensity sessions. The J-shaped curve primarily reflects acute permeability responses to single exercise bouts, whereas SCFA production represents a longer-term adaptive outcome. Source: The authors.)

The relationship between exercise and intestinal barrier integrity is not linear. A substantial body of evidence indicates that regular moderate exercise reinforces the intestinal barrier, whereas prolonged, high-intensity, or exhaustive exercise may transiently increase intestinal permeability—a phenomenon commonly referred to as “exercise-induced intestinal hyperpermeability” or “exercise-associated gut leakiness” [29,45]. This biphasic pattern is most appropriately described by a J-shaped dose–response curve: low exercise doses produce minimal effects, moderate doses confer maximal protection, whereas very high doses shift the balance toward detriment [29,38] (Figure 2).

Moderate-intensity continuous training performed for 30–60 min per session, 3–5 days per week, has been consistently demonstrated to improve intestinal barrier integrity [20,37]. In contrast, acute high-intensity or prolonged endurance exercise, such as marathon running, consistently increases intestinal permeability, as assessed by the lactulose/mannitol ratio or plasma I-FABP levels [29]. The mechanisms underlying this acute barrier disruption include splanchnic hypoperfusion, hyperthermia, enhanced sympathetic drive, and mechanical stress [40,58].

In addition to hemodynamic and mechanical factors, the magnitude of exercise-induced hyperthermia has been shown to independently correlate with increased intestinal permeability and altered tight junction gene expression in animal models [64]. This suggests that thermal stress, particularly during exercise in hot environments, may exacerbate barrier disruption through heat shock response pathways.

The reduction in splanchnic blood flow during intense exercise leads to intestinal hypoxia, which can compromise epithelial barrier integrity through hypoxia-inducible factor (HIF)-dependent pathways and ATP depletion. This ischemic insult, followed by reperfusion injury upon recovery, generates reactive oxygen species that further disrupt tight junction complexes and may facilitate bacterial translocation. In severe cases, especially during extreme endurance events, these disturbances can manifest as gastrointestinal symptoms including transient bleeding and diarrhea, as comprehensively reviewed by Costa et al. [47]. These acute effects are typically self-limiting in healthy individuals but may pose greater risks in those with pre-existing intestinal vulnerability. Trained athletes exhibit a higher threshold for exercise-induced intestinal permeability compared with untrained individuals, which is attributable to adaptive responses such as enhanced splanchnic collateral circulation and upregulation of heat shock proteins [32,38].

Based on the J-shaped curve, the following practical recommendations can be made [29,58]: (1) For the general healthy population, moderate-intensity aerobic exercise—such as brisk walking, jogging, or cycling—is recommended for 30–60 min per session, 3–5 times per week [20]; (2) Individuals with pre-existing intestinal diseases should begin at a lower intensity (40–50% of maximal oxygen uptake, VO_2_max) and shorter duration (20–30 min per session), with gradual progression under medical supervision [65]; (3) Prolonged (>90 min) high-intensity (>70% of VO_2_max) exercise should be avoided, particularly in hot environments where adequate hydration and heat acclimatization are not ensured [58]; (4) Athletes may mitigate exercise-induced intestinal hyperpermeability through heat acclimatization, carbohydrate loading, and supplementation with probiotics or glutamine [66].

## 3. Therapeutic Potential of Exercise in the Prevention and Management of Intestinal Diseases

Given the compelling evidence that moderate exercise enhances intestinal barrier integrity, attenuates inflammation, and modulates the gut microbiota [15,21,36], exercise may exert therapeutic or preventive effects in a spectrum of intestinal diseases characterized by barrier dysfunction and chronic inflammation [1,9].

### 3.1. Inflammatory Bowel Disease

Inflammatory bowel disease (IBD), which comprises Crohn’s disease and ulcerative colitis, is a chronic relapsing–remitting disorder driven by an aberrant immune response to the gut microbiota in genetically susceptible individuals [59]. Intestinal barrier dysfunction is both an early event in the pathogenesis of IBD and a perpetuating factor in its persistence [1].

In animal experiments, DSS-induced colitis mouse models subjected to 4 weeks of voluntary wheel running exhibited significant reductions in disease activity index, colonic mucosal erosion, and inflammatory cell infiltration, along with preserved colonic expression of ZO-1 and occludin [36]. Similarly, in 2,4,6-trinitrobenzene sulfonic acid (TNBS)-induced colitis rat models, 6 weeks of treadmill exercise alleviated the severity of colitis and restored colonic tight junction protein expression [36].

In clinical studies, a systematic review encompassing 12 studies with a total of 656 IBD patients found that moderate-intensity exercise was closely associated with reduced disease activity, improved quality of life, and decreased fatigue scores [12]. In a randomized controlled trial of 48 patients with mild-to-moderate ulcerative colitis, a 12-week walking program (30 min/day, 3 days/week) significantly reduced the Simple Clinical Colitis Activity Index and fecal calprotectin levels [65]. A large prospective cohort study of 1613 IBD patients also reported that higher levels of physical activity were associated with a lower risk of disease flares during 6 months of follow-up [12]. Overall, moderate exercise appears to be safe and beneficial for patients with IBD in remission or with mild-to-moderate disease [65]. Furthermore, exercise training has been shown to reduce inflammatory marker levels and improve physical function in IBD patients [67]. Systematic reviews and meta-analyses have further corroborated that physical activity is significantly associated with a lower risk of developing IBD [68].

### 3.2. Irritable Bowel Syndrome

Irritable bowel syndrome (IBS) is a functional gastrointestinal disorder characterized by chronic abdominal pain, bloating, and altered bowel habits [69]. Many IBS patients exhibit low-grade mucosal inflammation, altered intestinal permeability, and gut dysbiosis [12].

A systematic review and meta-analysis encompassing 10 randomized controlled trials with a total of 686 IBS patients demonstrated that exercise interventions significantly improve overall symptom severity, abdominal pain, bloating, and quality of life in IBS [69]. A recent trial in IBS patients stratified by body mass index further demonstrated that a structured walking program not only alleviated symptoms but also improved intestinal barrier integrity, reinforcing the therapeutic potential of exercise in this population [70]. In a 12-week randomized controlled trial of 102 IBS patients, a walking program (30 min/day, 3 days/week) reduced the IBS symptom severity score by 42%, compared with only 18% in the usual care group, and 57% of patients in the exercise group achieved the criterion of “adequate relief” [69]. The beneficial effects of exercise therapy on symptoms and quality of life in IBS patients have been confirmed by multiple randomized controlled trials [71]. The underlying mechanisms involve the alleviation of stress and anxiety through modulation of the hypothalamic–pituitary–adrenal (HPA) axis and vagal tone, enhancement of gastrointestinal motility, reduction in low-grade inflammation, and regulation of the gut microbiota [12,28].

### 3.3. Metabolic Syndrome-Associated Intestinal Barrier Injury

Obesity, type 2 diabetes mellitus, and non-alcoholic fatty liver disease are all characterized by chronic low-grade inflammation, which arises in part from increased intestinal permeability and subsequent lipopolysaccharide translocation—a state termed “metabolic endotoxemia” [11,59].

In high-fat-diet-induced obese mice, 12 weeks of voluntary wheel running restored colonic expression of ZO-1 and occludin, reduced serum lipopolysaccharide levels, and enriched Akkermansia muciniphila [49]. A randomized controlled trial of 60 obese adults reported that 12 weeks of moderate-intensity aerobic exercise reduced the lactulose/mannitol ratio by 22% and plasma lipopolysaccharide by 31%, and these changes were closely correlated with improvements in insulin resistance and inflammatory markers [11]. In a study of 45 patients with NAFLD, combined aerobic and resistance training similarly improved intestinal permeability and reduced hepatic fat content [59] (Table 3).

### 3.4. Other Intestinal Diseases and Future Directions

Preliminary evidence suggests that exercise may hold therapeutic potential in celiac disease, NSAID-associated enteropathy, and colorectal cancer survivors; however, further studies are warranted to validate these findings [1,24].

Despite the encouraging findings from preclinical and clinical studies, the therapeutic potential of exercise for intestinal diseases should be interpreted with appropriate caution. The existing clinical evidence is constrained by small sample sizes, short intervention durations, heterogeneous patient populations, and a lack of standardized exercise protocols [65,69,72]. Moreover, most human studies have relied on indirect biomarkers of intestinal permeability rather than direct functional or histological assessments of barrier integrity [1]. Consequently, while exercise holds promise as an adjunctive non-pharmacological strategy for symptom relief and barrier protection, its role as a definitive therapeutic intervention for intestinal diseases awaits confirmation through large-scale, long-term randomized controlled trials with standardized outcome measures and, where feasible, direct assessments of intestinal barrier function [12].

## 4. Comprehensive Comparison of Different Exercise Intervention Protocols on Intestinal Barrier Integrity

### 4.1. Exercise Modalities

Moderate-intensity continuous training (MICT) is currently the most well-established exercise modality, exerting potent barrier-protective effects through a combination of mechanisms, including upregulation of tight junction proteins, suppression of NF-κB-mediated inflammation, activation of Nrf2-driven antioxidant defenses, enhancement of short-chain fatty acid production, and promotion of myokine secretion [20,37]. High-intensity interval training (HIIT) also demonstrates barrier-protective effects with chronic adaptation; however, acute HIIT sessions may induce transient intestinal hyperpermeability and should therefore be applied with caution [29,55]. The evidence for resistance training is relatively weaker, with its effects primarily mediated through improvements in myokine profiles and insulin sensitivity [30]. Yoga is primarily indicated for symptom relief, exerting its effects through stress reduction, enhanced vagal tone, and suppression of inflammation [28,69]. In terms of tolerability in patients with intestinal conditions, MICT and yoga are generally well tolerated, HIIT shows moderate tolerability, whereas resistance training requires caution to avoid Valsalva maneuvers.

It should be noted, however, that direct comparisons between different exercise modalities are inherently constrained by substantial heterogeneity across the studies reviewed [29,30,36,53]. The cited investigations vary considerably in exercise intensity, session duration, intervention period, participant characteristics (e.g., age, baseline fitness level, disease status, and medication use), and outcome measures, which range from the lactulose/mannitol ratio and plasma I-FABP to transepithelial electrical resistance and tight junction protein expression [29,55]. These methodological differences preclude definitive quantitative conclusions regarding the superiority of any single modality. Accordingly, the comparative findings presented herein should be interpreted as qualitative trends rather than quantitative evidence, and future studies employing standardized protocols with head-to-head comparisons within the same experimental framework are urgently needed to establish more robust evidence [30].

### 4.2. Exercise Intensity, Frequency, and Dose

The optimal intensity range for improving intestinal barrier function is 50–70% of maximal oxygen uptake (VO_2_max), which corresponds to 65–80% of maximal heart rate [20,37]. Higher exercise frequency (3–5 days per week) confers greater benefits, whereas frequencies of 6 or more days per week provide no additional advantage [30]. The minimal effective dose is 20–30 min per session; however, exercise exceeding 90 min at moderate intensity or 60 min at high intensity increases the risk of barrier disruption, particularly in untrained individuals [29,58]. For subjective perception of exercise intensity, it is recommended to maintain a rating of 12–14 on the Borg Rating of Perceived Exertion (RPE) scale (Table 4).

Practical Recommendations for the General Healthy Population:

Aerobic exercise (walking, jogging, cycling) should constitute the primary modality, supplemented with resistance training 2–3 times per week, with yoga as an adjunctive option [20,30,69]. Intensity should be controlled at 50–70% of VO_2_max (corresponding to a Borg RPE of 12–14) [20], with a frequency of 3–5 days per week [30], and a duration of 30–60 min per session [20]. For deconditioned individuals or those with active intestinal diseases, exercise should be initiated at a lower intensity and shorter duration, with gradual progression over 4–8 weeks [65].

For healthy adults, aerobic exercise (e.g., brisk walking, jogging, or cycling) is recommended at an intensity of 50–70% of VO_2_max, for 30–60 min per session, 3–5 times per week, while avoiding prolonged (>90 min) high-intensity exercise, particularly in hot environments [20,30]. For patients with IBD in remission or with mild-to-moderate disease, walking, cycling, or swimming may be initiated at 20–30 min per session, 2–3 times per week, at low-to-moderate intensity, with exercise avoided during active flares and medical consultation advised [65]. For patients with IBS, moderate-intensity aerobic exercise for 20–45 min per session, 3–5 times per week is recommended, with yoga as an adjunctive option; however, it should be noted that high-intensity exercise may exacerbate symptoms in some patients [69]. For patients with NAFLD, obesity, or type 2 diabetes, moderate-intensity aerobic exercise for 30–60 min per session, 3–5 times per week is recommended, supplemented with resistance training 2–3 times per week, with adequate hydration ensured [11,59]. For athletes and endurance exercisers, heat acclimatization, carbohydrate loading, and supplementation with probiotics or glutamine may help mitigate exercise-induced intestinal hyperpermeability, along with monitoring for signs of gastrointestinal distress [66].

These recommendations represent general guidelines based on the current evidence. However, individual variability in baseline gut microbiota composition, genetic background, age, sex, and disease status may substantially influence the barrier response to exercise, underscoring the need for personalized exercise prescriptions—a direction we elaborate further in Section 6.3.

## 5. From Intestinal Barrier to Systemic Health: Integration of the Gut–Liver Axis and Gut–Brain Axis in Exercise

### 5.1. Gut–Liver Axis

The liver receives approximately 70% of its blood supply via the portal vein, making it the first organ to encounter gut-derived products [59]. When intestinal barrier integrity is compromised, lipopolysaccharide and other microbial products translocate into the portal circulation, activate Kupffer cells through TLR4, and thereby promote hepatic steatosis, fibrosis, and insulin resistance [11,59].

Regular moderate exercise has been shown to improve liver injury markers and reduce hepatic fat content in patients with NAFLD and ALD, with part of these effects mediated through exercise-induced improvements in intestinal barrier function [59]. In high-fat-diet-induced NAFLD mouse models, 12 weeks of voluntary wheel running not only reduced hepatic steatosis and serum alanine aminotransferase (ALT) levels, but also decreased portal lipopolysaccharide levels, and the reduction in portal LPS was strongly correlated with the improvement in steatosis [49]. Administration of an intestine-restricted TLR4 inhibitor abolished the hepatoprotective effects of exercise, further confirming the critical role of gut-derived lipopolysaccharide signaling in this process [11]. In human studies, a 12-week randomized controlled trial in patients with NAFLD demonstrated that moderate-intensity aerobic exercise reduced hepatic triglyceride content by approximately 28%, serum ALT by approximately 35%, and plasma lipopolysaccharide by approximately 32%, with changes in LPS independently correlated with changes in hepatic fat [59]. Furthermore, exercise modulates bile acid metabolism, increasing secondary bile acids and activating hepatic farnesoid X receptor (FXR), thereby suppressing gluconeogenesis, improving insulin sensitivity, and concurrently enhancing intestinal tight junction expression [59]. Combined dietary and exercise interventions have been validated as effective non-pharmacological therapeutic strategies for metabolic-associated fatty liver disease [73]. Exercise may also influence cardiometabolic diseases through modulation of the gut microecology [74].

### 5.2. Gut–Brain Axis

Intestinal barrier dysfunction and the consequent metabolic endotoxemia have been implicated in the pathogenesis of depression, anxiety, and other neuropsychiatric disorders [12]. Exercise exerts potent antidepressant effects through multiple neurobiological mechanisms, and emerging evidence suggests that exercise-induced improvements in intestinal barrier integrity may contribute to these mood-regulating effects [58].

In the chronic unpredictable mild stress (CUMS) rat model, 8 weeks of treadmill exercise increased colonic expression of ZO-1 and occludin, reduced serum lipopolysaccharide levels, and ameliorated depressive-like behaviors [43], whereas gut sterilization abolished the antidepressant effects of exercise [12]. In human studies, a 12-week moderate-intensity aerobic exercise intervention in 60 adults with mild-to-moderate depression reduced plasma lipopolysaccharide by approximately 24% and the lactulose/mannitol ratio by approximately 18%, and changes in LPS were significantly correlated with reductions in Beck Depression Inventory scores (r = 0.58). Mediation analysis revealed that the effects of exercise on depression were partially mediated by the reduction in LPS, with a mediated proportion of approximately 41% [12].

The short-chain fatty acids enriched by exercise—particularly butyrate—can cross the blood–brain barrier, inhibit histone deacetylases, enhance neuroplasticity, and reduce neuroinflammation [5,7]. Additionally, exercise has been shown to improve intestinal barrier function and reduce neuroinflammation in preclinical models of Parkinson’s disease, further supporting the role of the gut–brain axis in exercise-mediated neuroprotection [75]. Butyrate can also directly inhibit indoleamine 2,3-dioxygenase, shunting tryptophan metabolism away from neurotoxic kynurenine derivatives and toward serotonin synthesis [58]. Emerging evidence indicates that exercise-induced modulation of the kynurenine pathway, mediated in part by gut microbiota-derived metabolites, may represent a key mechanism linking physical activity to mood enhancement and fatigue resistance [76]. The vagus nerve is also indispensable for certain antidepressant effects of exercise—subdiaphragmatic vagotomy abolishes exercise-induced reductions in depressive-like behaviors [28]. Exercise influences mental health through modulation of the gut microbiota–gut–brain axis, with mechanisms involving neurotransmitter synthesis, regulation of inflammation, and alterations in neuroplasticity [77].

### 5.3. Integration and Clinical Implications

The gut–liver axis and the gut–brain axis are closely intertwined, with mutual interactions and overlapping mechanisms [12,59]. By restoring intestinal barrier integrity, exercise has the potential to concurrently improve both hepatic and mental health outcomes [17]. Assessment of intestinal permeability—using markers such as the lactulose/mannitol ratio, plasma lipopolysaccharide-binding protein, and I-FABP—may serve as useful biomarkers for monitoring the efficacy of exercise interventions in patients with hepatic or neuropsychiatric disorders [1].

## 6. Discussion

This review provides a comprehensive narrative synthesis of the molecular mechanisms by which exercise regulates intestinal barrier integrity and evaluates the therapeutic potential of different exercise interventions [17,20,21,22,32,55]. Several key findings emerge from this synthesis. First, regular moderate exercise enhances intestinal barrier function through multiple interconnected pathways, including upregulation of tight junction proteins [20,32], suppression of TLR4/NF-κB-mediated inflammation [21], activation of Nrf2-driven antioxidant defenses [22], and enrichment of short-chain fatty acid-producing gut microbiota [7,55]. Second, exercise induces a complex “peripheral–gut dialogue” mediated by myokines and vagal cholinergic signaling [25,26,28]. Third, the relationship between exercise and intestinal barrier function follows a J-shaped dose–response curve, whereby moderate exercise confers robust protection, whereas prolonged high-intensity exercise transiently increases permeability [29,38]. Fourth, exercise has exhibited therapeutic potential in intestinal barrier injury associated with IBD, IBS, and metabolic syndrome [11,65,69]. Fifth, exercise-induced improvements in intestinal barrier function translate into systemic health benefits via the gut–liver axis and the gut–brain axis [12,59].

### 6.1. Limitations of the Current Evidence

It should be noted that the present work is a narrative review and does not follow formal systematic review methodology. No prespecified search strategy, standardized study selection criteria, or formal risk-of-bias assessment was performed. The synthesis presented herein is therefore qualitative and interpretive in nature, reflecting a comprehensive but non-systematic integration of the available literature.

The field currently faces several limitations. First, the predominance of animal models and the translational gap from animals to humans remain significant challenges [36,49]. Second, the substantial heterogeneity in exercise protocols—including modality, intensity, frequency, and duration—makes direct comparisons across studies difficult [30,53]. Third, there is a lack of standardized, validated, and non-invasive biomarkers for assessing intestinal permeability [1]. Fourth, interindividual variability is often overlooked [9]. Fifth, the majority of studies are short-term in nature, with a notable paucity of long-term follow-up data [12]. Sixth, the potential for publication bias should also be acknowledged [29]. Seventh, the limitations of human studies deserve particular emphasis. The available clinical trials are characterized by small sample sizes, short follow-up periods (predominantly 12 weeks or less), and a conspicuous absence of multicenter designs [65,72]. Subgroup analyses stratified by age, sex, disease duration, baseline microbiota composition, or genetic background are rarely performed, limiting the generalizability of findings [1,31]. Moreover, there is currently no standardized exercise prescription guideline for routine gastroenterology clinical practice, which constrains the translation of research findings into actionable clinical recommendations [30]. The development of such guidelines, grounded in robust dose–response data, represents a critical unmet need in the field.

### 6.2. Controversies and Unresolved Questions

The major controversies currently under debate include: whether exercise is invariably beneficial to the gut—i.e., the existence of the J-shaped curve [38]; which exercise prescription is optimal for gut health [20]; whether changes in the gut microbiota are causes or consequences of exercise effects [15,16]; and whether exercise can replace pharmacological interventions—with current evidence supporting its role only as an adjunctive therapy [65,69].

A further unresolved question pertains to the causal nature of the associations observed between exercise, pathway activation, and barrier improvement. The majority of studies to date are correlative, demonstrating that exercise, upregulation of protective pathways, and enhanced barrier function co-occur [20,21,22]. However, whether these pathways are the primary mediators of exercise-induced barrier protection, or merely epiphenomena, remains uncertain. Pathway-specific inhibitor studies, genetic knockout models, and pharmacological blockade experiments—while feasible in animals—are ethically and logistically challenging in humans [32,47]. This evidentiary gap limits the strength of causal inferences that can be drawn regarding the mechanistic hierarchy and relative contributions of each pathway to the observed barrier-protective effects. Addressing this limitation will require innovative study designs, including longitudinal intervention–washout paradigms and within-subject repeated-measures approaches, to better establish temporal and causal relationships.

### 6.3. Future Directions

Future research should prioritize the following areas: (1) conducting large-scale, long-term randomized controlled trials with standardized outcome measures [12]; (2) integrating multi-omics data to predict individual responses and advance precision exercise medicine [9]; (3) utilizing tools such as endoscopic biopsy or ingestible capsules to conduct mechanistic studies in humans [1]; (4) exploring the synergistic effects of combining exercise with probiotics, prebiotics, or dietary fiber interventions [66]; (5) developing validated, non-invasive biomarkers for clinical use [1]; and (6) translating research findings into clinical practice guidelines [30].

In particular, two directions warrant expanded discussion:

#### 6.3.1. Precision Personalized Exercise Medicine

The substantial interindividual variability in gut microbiota composition, baseline intestinal permeability, inflammatory status, and genetic background observed across studies [15,29,31] underscores the need for individualized approaches to exercise prescription. Recent multi-omics and population-stratification studies have begun to identify host and microbial factors that modulate the barrier response to exercise [15,31,52]. However, the current evidence base is insufficient to define specific protocols for distinct patient subgroups. Future research should aim to develop stratified exercise prescriptions based on validated intestinal permeability biomarkers and individual phenotypic characteristics, moving beyond uniform generic protocols to maximize therapeutic benefits while minimizing the risk of exercise-induced hyperpermeability in susceptible individuals [9,30,31].

#### 6.3.2. Synergistic Interventions: Exercise Combined with Probiotics/Prebiotics

Emerging evidence from athlete, IBD, and obese population studies suggests that probiotic supplementation may alleviate high-intensity exercise-induced gut leakiness, while exercise may in turn enhance probiotic colonization efficiency [23,50,55,66]. The combination of exercise with probiotics or prebiotics may confer additional benefits over exercise alone in terms of SCFA production, tight junction protein upregulation, and inflammatory suppression [7,23,50]. However, the current evidence is largely preliminary, with limited sample sizes and heterogeneous intervention protocols. Rigorous, well-controlled trials are needed to establish whether combined interventions yield clinically meaningful additive effects and to identify optimal co-intervention strategies for specific patient populations [66].

Beyond probiotics, nutritional supplementation such as alanyl-glutamine has been shown to protect intestinal barrier function against acute exhaustive exercise in trained rats by upregulating ZO-1 and occludin expression [72]. This provides further support for exploring combined exercise–nutritional strategies to mitigate exercise-induced intestinal hyperpermeability [78].

## 7. Conclusions

Moderate exercise—comprising aerobic activity at 50–70% of maximal oxygen uptake, performed for 30–60 min per session, 3–5 days per week—enhances intestinal barrier integrity through upregulation of tight junction proteins, suppression of NF-κB, activation of Nrf2, and remodeling of the gut microbiota and short-chain fatty acid profile [12]. Exercise induces a “peripheral–gut dialogue” mediated by myokines (irisin, IL-6, BDNF) and vagal cholinergic signaling [25,26,28]. The dose–response relationship follows a J-shaped curve: moderate exercise protects the gut, whereas excessive exercise leads to transient “exercise-induced intestinal hyperpermeability” [29,38]. Exercise has shown preliminary therapeutic potential as an adjunctive, non-pharmacological auxiliary intervention in IBD, IBS, and metabolic endotoxemia; however, these findings require confirmation in larger, well-controlled human studies before definitive clinical recommendations can be made [11,65,69]. The exercise-induced improvements in intestinal barrier function confer simultaneous benefits to the liver (via the gut–liver axis) and the brain (via the gut–brain axis) [12,59]. Important limitations in the current evidence exist; future research should prioritize the development of precision exercise medicine, long-term trials, and mechanistic studies in humans [1,30,72].

Exercise is a potent, convenient, and cost-effective non-pharmacological strategy for maintaining intestinal barrier integrity [17]. When prescribed appropriately, it can reinforce the first line of defense in the gut, alleviate systemic inflammation, and contribute to the prevention and management of both intestinal and systemic diseases [15,21].

## Figures and Tables

**Table 1:** Representative Studies on the Effects of Exercise on Tight Junction Protein Expression.

Study	Model	Exercise Protocol Key	Findings
Ghosh et al., 2012 [36]	High-fat diet-fed mice	Voluntary wheel running, 12 weeks	↑ ZO-1 and occludin in small intestine and colon
van Wijck et al., 2011 [19]	Marathon runners	Single marathon run	↑ I-FABP, ↓ serum occludin
Pasini et al., 2019 [37]	Patients with type 2 diabetes	Chronic exercise intervention	Improved gut microbiota composition and barrier function

*Note: ↑ indicates an increase; ↓ indicates a decrease.*

**Table 2:** Key Studies on Exercise, Gut Microbiota, and SCFAs.

Study	Population/Model	Exercise Protocol	Key Findings
HIGH-EX vs. MOD-EX pooled analysis (2025) [55]	Patients with metabolic syndrome (*n* = 113)	12 weeks of HIIT vs. MICT + resistance training	HIIT only: total SCFAs +30%; lactate correlated with SCFA increase (r = 0.68)
Muguerza-Rodríguez et al., 2025 [60]	Type 2 diabetes (systematic review)	Structured exercise	↑ Faecalibacterium, Veillonella, Lachnospira, Bifidobacterium
Kasimu et al., 2026 [61]	Stroke rats (tMCAO)	Treadmill exercise preconditioning, 2 weeks	↑ Gut microbial diversity, ↑ Lactobacillus
Bianco A et al., 2024 [62]	IBS patients (BMI-stratified)	Fitwalking program	↑ Physical capacity and intestinal barrier integrity

*Note: ↑ indicates an increase.*

**Table 3:** Key Clinical Studies on the Interventional Effects of Exercise in Intestinal Diseases.

Disease	Study	Sample Size	Exercise Protocol	Main Outcomes
IBD	Klare et al., 2015 [65]	48 UC patients	Walking 30 min/day, 3 days/week, 12 weeks	↓ SCCAI, ↓ fecal calprotectin
IBS	Johannesson et al., 2011 [68]	102 IBS patients	Walking 30 min/day, 3 days/week, 12 weeks	↓ IBS-SSS by 42%
NAFLD	Pooled RCT data [11,59]	60 patients	Aerobic exercise 45 min/day, 5 days/week, 12 weeks	↓ Hepatic fat, ↓ plasma LPS

*Note: ↓ indicates a decrease.*

**Table 4:** Summary of the Effects of Different Exercise Protocols on Intestinal Barrier Outcomes.

Exercise Parameter	Specific Category	Effect on Intestinal Barrier	Recommendation Level
Modality	Moderate-intensity continuous training (MICT) [20]	Strong protection	Strongly recommended
	High-intensity interval training (HIIT) [55]	Moderate protection (chronic adaptation)	Cautiously recommended HIIT
	Resistance training [30]	Mild protection	Adjunctive
	Yoga [69]	Symptom improvement	Adjunctive
Intensity	Moderate (50–70% VO_2_max) [20]	Optimal protection	Strongly recommended
	High (>75% VO_2_max, >60 min) [29]	Transiently detrimental	Avoid
Frequency	3–5 days/week [30]	Optimal protection	Strongly recommended
Duration	30–60 min/session [20]	Optimal protection	Strongly recommended
	>90 min at moderate intensity [58]	Increased risk of leaky gut	Avoid

Comparisons in this table are qualitative summaries of individual study trends rather than quantitative meta-analytic comparisons, owing to substantial heterogeneity in intervention protocols and outcome measures across the included studies [29,30,53].

## Data Availability

No new data were created or analyzed in this study. Data sharing is not applicable.
